# Sex-related analgesic responses to photobiomodulation therapy in rats with neuropathic pain

**DOI:** 10.1007/s10103-026-05046-5

**Published:** 2026-09-25

**Authors:** Natasha Farias Marques, Regina Paula Bonifacio, Juliana Mota Jesus, Maria Eduarda Rodrigues Santos, Matias Cardoso Grande, Hilária Conda Quimuanga, Margarida I C Goncalves, Danielle Paula F B Silva, Marucia Chacur

**Affiliations:** 1 Institute of Biomedical Science, Department of Anatomy, Universidade de São Paulo, São Paulo, Brazil; 2 Medical School, University of Caxias Do Sul, Caxias do Sul, Brazil

**Keywords:** Neuropathic pain, Sex differences, Estrous cycle, Photobiomodulation therapy

## Abstract

Neuropathic pain is associated with sensory abnormalities and remains difficult to treat. Photobiomodulation therapy (PBMT) has emerged as a non-pharmacological approach with analgesic potential; however, sex-related differences in treatment responses remain poorly understood. We investigated the effects of PBMT in male and female rats subjected to chronic constriction injury (CCI) of the sciatic nerve. Animals received infrared PBMT administered three times per week during ten sessions, and nociceptive responses were assessed using mechanical hyperalgesia and tactile allodynia throughout treatment. CCI induced significant nociceptive hypersensitivity in both sexes. PBMT progressively increased withdrawal thresholds and reduced pain-like behaviors. Females exhibited greater relative recovery in both mechanical hyperalgesia and tactile allodynia, whereas males maintained higher absolute nociceptive thresholds throughout the experimental period. Estrous cycle analysis revealed reduced pain sensitivity during metestrus compared with estrus and proestrus. These findings demonstrate that PBMT effectively attenuates neuropathic pain-like behaviors in both sexes and suggest that biological sex and hormonal status may influence therapeutic responses. The results support the inclusion of sex as a biological variable in preclinical photobiomodulation studies.

**Supplementary Information:**

The online version contains supplementary material available at https://doi.org/10.1007/s10103-026-05046-5.

## Introduction

Neuropathic pain results from injury or dysfunction of the somatosensory nervous system and is characterized by hyperalgesia, allodynia, and persistent sensory disturbances [1–3]. Despite available pharmacological treatments, many patients experience inadequate pain relief and adverse effects, highlighting the need for alternative therapeutic approaches [4].

PBMT has gained attention as a non-invasive intervention capable of modulating mitochondrial activity, reducing inflammation, and promoting tissue repair. Experimental studies have demonstrated beneficial effects of PBMT in neuropathic pain models; however, most investigations have been conducted predominantly in male animals [5–8].

Evidence from the literature supports the beneficial effects of PBMT on neuropathic pain like behaviors in different models of peripheral nerve injury, including CCI of the sciatic nerve, sciatic nerve crush, diabetic neuropathy, and chemotherapy-induced neuropathy [9–13]. Reported beneficial effects include reductions in mechanical allodynia and thermal hyperalgesia, together with improvements in nerve regeneration and tissue repair. Although the mechanisms are not yet fully understood, current evidence suggests that PBMT promotes analgesia through the modulation of mitochondrial metabolism, enhancement of ATP synthesis, reduction of oxidative stress, regulation of pro- and anti-inflammatory cytokines, attenuation of glial activation, and modulation of nociceptive signaling pathways [14–16]. These findings support PBMT as a promising non-pharmacological strategy for neuropathic pain while highlighting the need for further mechanistic investigations.

Previous studies from our group have demonstrated that PBMT effectively reverses neuropathic pain in animal models, although its underlying mechanisms have not yet been fully elucidated. In rats with diabetic neuropathy, PBMT reduces the expression of the receptor for advanced glycation end-products (RAGE) and nuclear factor kappa B (NF-κB) in the peripheral nervous system, while suppressing tumor necrosis factor-alpha (TNF-α) expression in the central nervous system [17]. Additionally, PBMT increases the levels of the anti-inflammatory cytokine interleukin-10 (IL-10) in both the peripheral and central nervous systems of diabetic rats. In a rat model of CCI, PBMT reduced the expression of substance P and transient receptor potential vanilloid 1 (TRPV1) in the dorsal root ganglia (L4–L6) [12, 18]. Furthermore, immunoblotting and immunofluorescence analyses demonstrated a reduction in astrocyte activation within the dorsal root ganglia, suggesting that modulation of glial responses contributes to the analgesic effects of PBMT [19].

Growing evidence indicates that biological sex influences pain perception, immune responses, mitochondrial metabolism, and the efficacy of several analgesic interventions. Sex hormones modulate inflammatory pathways, glial activation, and mitochondrial function, all of which have been proposed as targets of PBMT. Sex differences are increasingly recognized as important determinants of pain processing and analgesic responses, with females generally exhibiting greater pain sensitivity and potentially different responses to therapeutic interventions due to hormonal and neuroimmune mechanisms [20–24]. Therefore, considering sex as a biological variable is important for improving the translational relevance of preclinical studies. Nevertheless, whether PBMT produces comparable or sex-dependent analgesic responses in neuropathic pain models remains insufficiently explored.

Despite the growing evidence supporting the analgesic effects of PBMT in neuropathic pain, its efficacy has been investigated predominantly in male animals, leaving potential sex-dependent responses largely unexplored. Addressing this gap is important for determining whether the beneficial effects of PBMT observed in preclinical studies are consistent across sexes and for improving the translational relevance of this therapeutic approach. In the present study, we therefore evaluated the effects of PBMT on neuropathic pain-like behaviors in male and female rats subjected to CCI injury. In female rats, we additionally examined nociceptive responses according to estrous cycle phase to characterize whether hormonal status contributes to variability in pain sensitivity.

## Methodology

### Animals

Male and female Wistar rats weighing between 180 and 220 g (approximately 2 months old) were used in all experiments. Male and female rats were housed separately, with five animals of the same sex per cage, under a controlled 12:12-h light/dark cycle and with free access to food and water. The cages used in our animal facility were larger than the standard cages routinely used for rats and accommodated a maximum of five animals per cage under the institutional housing conditions. All animals were handled in accordance with the principles and guidelines for the use of laboratory animals involving pain and nociception [25]. Male and female animals were distributed across the same experimental conditions, with *n* = 10 animals of each sex per group: (1) animals subjected to CCI without treatment (CCI + PBMT OFF), (2) sham-operated animals used as controls (Sham), and (3) animals subjected to CCI and treated with photobiomodulation (CCI + PBMT ON). Thus, males and females were analyzed as separate cohorts under identical experimental conditions.

### Estrous cycle assessment

Estrous cycle phases were evaluated daily between 8:00 and 10:00 a.m. by vaginal cytology, as previously described [26]. Monitoring began before surgery and continued throughout the experimental period until the end of the study. Vaginal smears were collected before each behavioral assessment to determine the estrous phase at every experimental time point. Smears were classified as proestrus (predominance of nucleated epithelial cells), estrus (predominance of cornified anucleated epithelial cells), metestrus (mixed population of nucleated epithelial cells, cornified cells, and leukocytes), or diestrus (predominance of leukocytes). For the analysis presented in Table 1, behavioral data from all female rats (*n* = 10–11, depending on the behavioral test) were pooled and grouped according to the estrous cycle phase to evaluate the influence of hormonal status on nociceptive thresholds. Analyses of PBMT treatment effects were performed separately using the predefined experimental groups.

### Chronic constriction of the sciatic nerve (CCI)

Neuropathic pain was induced using the CCI model described by Bennett and Xie [27]. Under isoflurane anesthesia, the sciatic nerve was exposed and loosely ligated with four chromic catgut sutures. Sham-operated animals underwent sciatic nerve exposure without ligation. Animals were randomly allocated to the experimental groups, and behavioral assessments were performed by an investigator blinded to group allocation. Animals were included according to the predefined experimental protocol, and no animals were excluded based on behavioral outcomes.

### Photobiomodulation treatment – PBMT

PBMT was initiated 14 days after CCI using an infrared laser device (IBRAMED, Brazil). Treatment was administered three times per week for ten sessions along the sciatic nerve. Sex-specific irradiation parameters were selected based on previous studies from our group demonstrating analgesic efficacy in male and female rats, respectively [12, 18]. Detailed laser parameters are provided in Supplementary Table S1. Animals assigned to the PBMT OFF groups underwent identical handling procedures with the laser device switched off.

### Behavioral test

To reduce stress, the rats were habituated to the test procedure the day before the experiment and again 45 min before behavioral assessment. Baseline behavioral measurements were obtained before any experimental procedure (BL). A second behavioral assessment was performed 14 days after CCI, immediately before the initiation of PBMT. Three additional behavioral assessments were performed once per week during the PBMT treatment period, after every three PBMT sessions. All behavioral tests were performed during the morning, at approximately the same time of day, to minimize potential circadian influences on nociceptive responses. Behavioral assessments were therefore performed at baseline, 14 days after CCI before first PBMT, and at three subsequent weekly time points during PBMT treatment (after 3, 6, and 9 PBMT sessions). Both tests were conducted by an investigator blinded to the experimental group assignment.

#### Mechanical hyperalgesia

To assess the animal’s pain sensitivity, the rat paw pressure test (Analgesy-Meter Ugo Basile^®^, Italy) was used, performed according to the method described by Randall and Sellito (1957) [28]. In this test, an increasing force in grams (16 g/s) was continuously applied to the right hind paw until withdrawal occurred. The withdrawal threshold was obtained from a single valid measurement at each experimental time point to avoid sensitization induced by repeated noxious stimulation.

#### Tactile Allodynia

The von Frey test was used to assess low-threshold mechanical pain thresholds, according to the method described by Chaplan (1994) [29]. For the allodynia assay, a logarithmic series of 10 von Frey filaments (Aesthesiometer Semmes Weinstein, Stoelting Co., USA) were applied to the middle of the plantar surface of the right hind paw for a maximum of 10 s to determine the threshold intensity of the stiffness stimulus required to elicit a paw withdrawal response. The filament capable of inducing paw withdrawal twice consecutively was considered as the force in grams required to induce the response (100% response).

### Statistical analysis

Statistical analyses and graphical representations of the data were performed using GraphPad Prism version 8.1 (GraphPad Software, San Diego, CA, USA). Data are presented as mean ± standard error of the mean (SEM). For comparisons involving measurements obtained at multiple time points from the same animals, two-way repeated-measures ANOVA was used, with treatment group and time as factors, followed by Tukey’s multiple-comparisons test when appropriate. For comparisons between male and female rats, two-way repeated-measures ANOVA was performed with sex and time as factors, followed by Tukey’s multiple-comparisons test. One-way ANOVA followed by the appropriate post hoc test was used for comparisons involving a single factor. Statistical significance was set at *P* < 0.05.

## Results

### Influence of estrous cycle in nociceptive thresholds

CCI surgery significantly reduced nociceptive thresholds, confirming the development of pain hypersensitivity as we can see in Table 1. Nociceptive responses varied across estrous cycle phases, particularly after the experimental protocol (Post). In mechanical hyperalgesia test, animals in metestrus exhibited higher nociceptive thresholds compared to estrus (*p* = 0.0309), indicating reduced pain sensitivity in this phase. Similarly, in the tactile allodynia test, metestrus was associated with higher thresholds compared to both proestrus (*p* = 0.0267) and estrus (*p* = 0.0206). No significant differences were observed for diestrus. Overall, these findings indicate that the estrous cycle modulates nociceptive sensitivity following CCI, with metestrus representing a phase of reduced pain sensitivity compared to other cycle stages (Table 1).

**Table 1:** Nociceptive thresholds across estrous cycle phases in females rats with CCI

Mechanical Hyperalgesia (g)			Tactile Allodynia (g)	
Estrous Cycle	Baseline (Mean ± SEM)	Post (Mean ± SEM)	Baseline (Mean ± SEM)	Post (Mean ± SEM)
Proestrus	58,3 ± 8,156	37,9 ± 2,81	7,25 ± 1,632	0,68 ** ± 0,1098
Estrus	58,1 ± 7,82	34,4 ±2,966	9,93 ± 0,8181	0,61# ± 0,04561
Metestrus	57 ±5,417	46* ±4,319	6,83 ± 0,4106	1,67±0,5354
Diestrus	65,3 ± 6,966	35 ± 1,331	9,3 ± 1,033	0,82 ± 0,1364

Nociceptive thresholds were assessed across estrous cycle phases (proestrus, estrus, metestrus, and diestrus) using the mechanical hyperalgesia and tactile allodynia tests expressed in grams (g). Data are expressed as mean ± SEM. Statistical analysis was performed using one-way ANOVA followed by Tukey’s post hoc test Sample size was n=11 at baseline and n=10 post-protocol due to the loss of one animal

*p= 0.0309: significant difference in metestrus compared to estrus (mechanical hyperalgesia)

**p= 0.0267: significant difference in metestrus compared to proestrus (tactile allodynia)

#p = 0.0206: significant difference in metestrus compared to estrus (tactile allodynia)

### Effect of PBMT treatment in mechanical hyperalgesia threshold

**Fig. 1:**
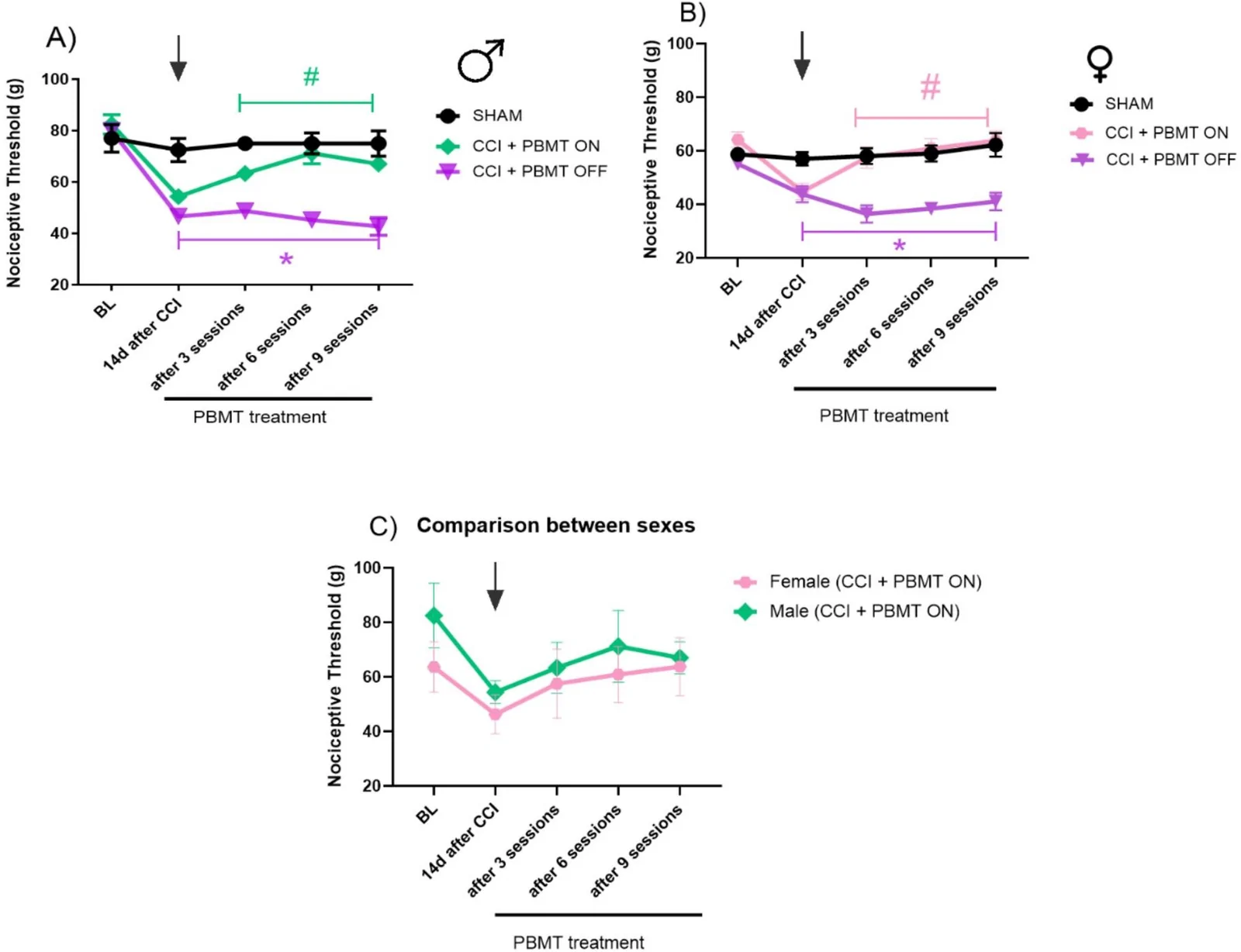
Effect of PBMT on mechanical hypersensitivity in male and female rats following CCI. The arrow indicates the onset of PBMT treatment. The y-axis represents mechanical withdrawal threshold (g), and the x-axis represents time. **A** Mechanical withdrawal thresholds in male rats. **B** Mechanical withdrawal thresholds in female rats. In both sexes, the CCI + PBMT ON group exhibited higher mechanical withdrawal thresholds than the CCI + PBMT OFF group from the second week of PBMT treatment onward. *N* = 10/group. *P < 0.05 vs. Sham group; #*P* < 0.05 vs. CCI + PBMT OFF group. **C** Direct comparison of absolute mechanical withdrawal thresholds between male and female rats in the CCI + PBMT ON groups. Data were analyzed using two-way ANOVA with sex and time as factors, followed by Tukey’s multiple-comparisons test. A significant main effect of sex was observed, indicating higher absolute thresholds in males; however, the sex × time interaction was not significant

Nociceptive thresholds were reduced following CCI surgery in both male and female rats, confirming the development of mechanical hypersensitivity (Fig. 1). In males, the CCI + PBMT ON group exhibited higher mechanical withdrawal thresholds than the CCI + PBMT OFF group from the second week of PBMT treatment onward, reaching an approximately 40.62% higher threshold at the end of the treatment period (Fig. 1A; F(8,127) = 5.198; *P* < 0.0001). A similar pattern was observed in females, with the CCI + PBMT ON group showing higher mechanical withdrawal thresholds than the CCI + PBMT OFF group from the second week onward, reaching an approximately 57% higher threshold at the end of the experiment (Fig. 1B; F(8,124) = 3.508; *P* = 0.001). These findings indicate that PBMT reduced mechanical hypersensitivity in both sexes.

In the direct comparison between sexes, male rats exhibited higher absolute mechanical withdrawal thresholds overall than female rats (Fig. 1C). However, because baseline differences in nociceptive thresholds were already present between sexes, absolute threshold values were not considered an appropriate measure of relative analgesic efficacy. Therefore, the higher absolute thresholds observed in males should not be interpreted as evidence of a greater analgesic effect of PBMT in this sex. Importantly, the comparison between sexes did not demonstrate a significant sex × time interaction, indicating no statistical evidence for a differential temporal response to PBMT between males and females.

### Effect of PBMT treatment in tactile allodynia

**Fig. 2:**
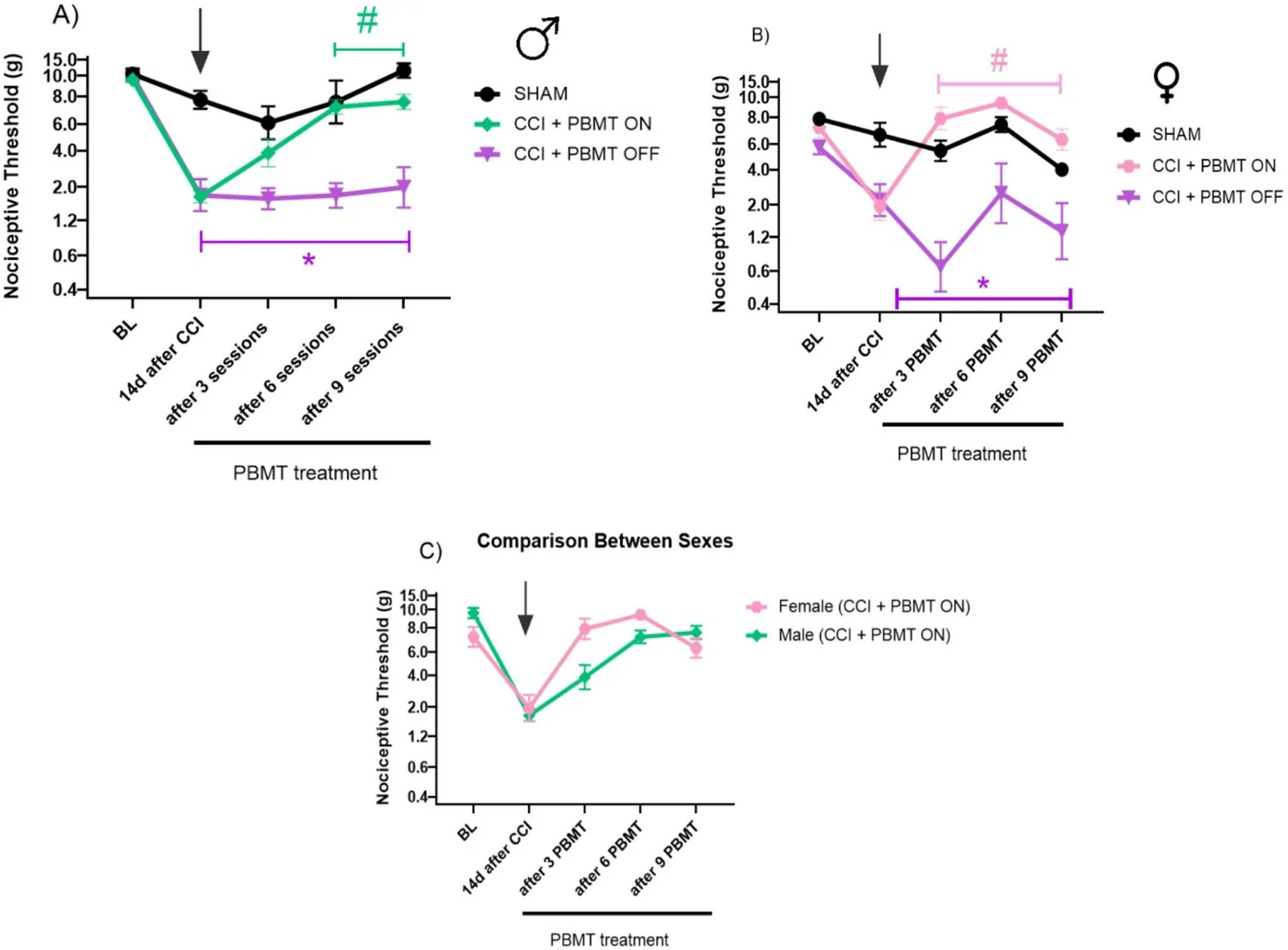
Effect of PBMT on tactile allodynia in male and female rats following CCI. The arrow indicates the onset of PBMT treatment. The y-axis represents nociceptive threshold (g), and the x-axis represents time after initiation of PBMT treatment. **A** Tactile allodynia in male rats (*n* = 7/group). **B** Tactile allodynia in female rats (*n* = 5/group). In both sexes, the CCI + PBMT ON group showed higher nociceptive thresholds than the CCI + PBMT OFF group during the treatment period. *P < 0.05 vs. Sham group; #*P* < 0.05 vs. CCI + PBMT OFF group. **C** Direct comparison of absolute nociceptive thresholds between male and female rats in the CCI + PBMT ON groups. Data were analyzed using two-way ANOVA with sex and time as factors, followed by Tukey’s multiple-comparisons test. A significant main effect of sex was observed, indicating higher absolute thresholds in males; however, the sex × time interaction was not significant

CCI surgery markedly reduced paw withdrawal thresholds in both male and female rats, confirming the development of tactile allodynia (Fig. 2). In male rats, the CCI + PBMT ON group showed higher nociceptive thresholds than the CCI + PBMT OFF group from the third week of PBMT treatment onward, reaching an approximately 122.23% higher threshold than the untreated group at the end of the treatment period (Fig. 2A). In female rats, the CCI + PBMT ON group showed higher nociceptive thresholds than the CCI + PBMT OFF group from the second week of treatment onward, reaching an approximately 238.45% higher threshold than the untreated group at the end of the experiment (Fig. 2B). These findings indicate that PBMT reduced tactile allodynia in both sexes.

In the direct comparison between sexes, female rats exhibited higher absolute nociceptive thresholds than males at the initial time points, whereas the difference between sexes became smaller over the course of treatment, with similar threshold values observed at the final time point (Fig. 2C). Because baseline differences in absolute nociceptive thresholds were observed between sexes, these absolute values were not interpreted as evidence of greater analgesic efficacy in either sex. Furthermore, the absence of a significant sex × time interaction would indicate that there was no statistical evidence for a differential temporal response to PBMT between males and females.

## Discussion

The inclusion of sex as a biological variable has become a major priority in preclinical pain research, given the growing evidence that males and females differ in pain processing, neuroimmune regulation, mitochondrial biology, and responses to analgesic therapies. Nevertheless, despite the increasing use of photobiomodulation therapy for neuropathic pain, whether its analgesic effects differ between sexes has remained largely unexplored. By directly comparing male and female rats, the present study helps address this important knowledge gap and provides evidence that PBMT attenuates neuropathic pain-like behaviors in both sexes, supporting further preclinical investigation of this therapeutic approach. This study demonstrates that PBMT attenuates neuropathic pain-like behaviors in both male and female rats following CCI. Improvements were observed in mechanical hyperalgesia and tactile allodynia, supporting previous evidence that PBMT exerts analgesic effects in neuropathic pain models [30–35].

Similar findings have been reported in CCI models treated with transcutaneous PBMT, in which the therapy significantly reduced mechanical allodynia whether administered before or after nerve injury [30].Considering that neuropathic pain remains difficult to treat and conventional pharmacological therapies often provide only partial symptom relief, these findings support further investigation of PBMT as a potential non-pharmacological therapeutic approach [33–35]. The biological plausibility of these analgesic effects is supported by previous studies suggesting that PBMT may influence mitochondrial function, oxidative stress, inflammatory responses, and neural repair [34]. Other experimental studies have also suggested that PBMT can modulate glial and neuroinflammatory pathways involved in central sensitization [33, 36, 37]. However, these mechanisms were not directly investigated in the present study and should therefore be considered possible explanations for the behavioral effects observed here.

An important finding was the presence of sex-related differences in treatment responses. Although both sexes benefited from PBMT, females exhibited greater relative recovery, whereas males maintained higher absolute withdrawal thresholds. These findings are consistent with accumulating evidence indicating that biological sex influences nociceptive processing, pain chronification, and responsiveness to analgesic interventions [33, 38–40]. Recent reviews suggest that sex differences in chronic pain extend beyond quantitative variations in pain sensitivity and instead involve distinct hormonal, neuronal, and neuroimmune mechanisms [35, 36, 38]. Consequently, the magnitude and pattern of PBMT-induced analgesia may be influenced by sex-specific biological pathways rather than differences in baseline nociceptive sensitivity alone [38, 41].

Previous experimental models of peripheral neuropathy further support the existence of sexually dimorphic pain responses, although the direction and magnitude of these differences vary depending on the experimental model, behavioral outcome, and time point evaluated. In the spared nerve injury (SNI) model, females develop mechanical and cold hypersensitivity earlier than males [42], whereas reviews of CCI models frequently report greater mechanical allodynia and delayed recovery in females [36]. Conversely, studies evaluating anti-inflammatory therapies have demonstrated greater therapeutic efficacy in males than females [43]. Therefore, the response pattern observed in the present study is not anomalous but rather consistent with an expanding body of literature demonstrating that analgesic efficacy depends on sex-specific biological mechanisms [40, 44, 45].

The estrous cycle analysis further supports the contribution of hormonal factors. Animals evaluated during metestrus displayed higher nociceptive thresholds than those in estrus, suggesting reduced pain sensitivity during this phase. Similar observations have been described in experimental pain studies, indicating that fluctuations in ovarian hormones influence both neuronal excitability and neuroimmune responses, although the estrous cycle alone does not fully explain sexual dimorphism in pain [41]. While some experimental studies report significant behavioral variations across estrous phases, others have found minimal associations between estrous stage and pain behavior, suggesting that hormonal influences are highly dependent on the experimental model and timing of assessment [42]. Thus, the present findings highlight the potential contribution of hormonal status to variability in nociceptive responses in female animals and support its consideration in future studies of analgesic interventions.

However, the interpretation of the estrous cycle-related findings should be considered in light of a methodological limitation. Although this approach allowed us to account for estrous cycle phase when interpreting nociceptive responses, repeated vaginal sampling and associated handling may represent a potential source of stress that could influence behavioral outcomes. Previous studies have suggested that repeated vaginal cytology may affect behavioral measures in rodents, although the extent to which this applies to nociceptive responses remains unclear. Therefore, we cannot exclude the possibility that repeated sampling contributed to variability in nociceptive thresholds in female rats. Future studies should consider experimental approaches that minimize repeated handling and vaginal manipulation when investigating sex- and estrous cycle-related differences in pain responses.

Sex differences in neuropathic pain have also been associated with differences in neuroimmune mechanisms. Previous studies have suggested a greater contribution of spinal microglial and P2 × 4/BDNF signaling in males, whereas adaptive immune mechanisms involving T lymphocytes may contribute more prominently to pain processing in females [45, 46]. Although these mechanisms were not directly investigated in the present study, they represent possible biological pathways that may contribute to the sex-related differences observed in PBMT responsiveness.

Interestingly, the magnitude of PBMT-induced analgesia differed according to the nociceptive modality assessed. While both mechanical hyperalgesia and tactile allodynia were attenuated, the pattern of recovery was not identical between sexes. The magnitude of PBMT-induced analgesia also differed according to the nociceptive modality assessed. Mechanical hyperalgesia and tactile allodynia involve partially distinct sensory processing mechanisms, including contributions from different populations of primary afferent fibers [35, 40, 47, 48]. Thus, the different behavioral patterns observed may reflect modality-dependent effects of PBMT; however, the underlying neural pathways were not directly assessed in the present study.

The use of different PBMT irradiation parameters for male and female animals in the present study was not arbitrary but was based on previous experimental findings from our group indicating sex-dependent differences in the antinociceptive response to photobiomodulation [30, 49]. These previous observations suggested that optimization of irradiation parameters may be necessary to achieve an effective analgesic response in each sex. Therefore, the parameters selected in the present study were based on sex-specific experimental evidence and were intended to maximize the therapeutic response rather than to introduce an additional experimental variable. Importantly, this approach should be considered in the context of the well-recognized photobiomodulatory dose-response relationship, in which treatment efficacy may be influenced by several interacting parameters, including wavelength, irradiance, energy density, exposure time, and treatment frequency [50–53].Experimental studies have demonstrated that changes in irradiance can substantially modify the analgesic effects of PBMT even when the delivered energy density is maintained, highlighting that energy density alone does not fully characterize the biological dose of light. Moreover, evidence from studies including both male and female subjects indicates that biological sex may influence the magnitude of the analgesic response to photobiomodulation, further supporting the need to consider sex when optimizing treatment parameters [50–53]. Thus, rather than assuming that a single irradiation protocol is universally optimal, these findings support a more individualized approach in which PBMT parameters are experimentally optimized according to biological sex and the characteristics of the pain model.

This consideration is particularly relevant in neuropathic pain, in which substantial heterogeneity exists in the development, maintenance, and temporal trajectory of pain between and within sexes [38, 54]. Sex-dependent differences in immune signaling, glial responses, hormonal regulation, neuronal excitability, and mitochondrial function may contribute to differences in both pain mechanisms and therapeutic responsiveness. Accordingly, the use of sex-specific PBMT parameters may represent an important methodological consideration when the objective is to achieve comparable therapeutic efficacy across sexes. Nevertheless, future studies should directly compare optimized and identical PBMT protocols in male and female animals to determine whether sex-dependent differences in treatment response persist when irradiation parameters are systematically controlled. Such studies would help distinguish differences attributable to biological sex from those arising from parameter-dependent effects and would contribute to the development of evidence-based, sex-informed PBMT protocols.

Importantly, the present study addresses a significant gap in the preclinical PBMT literature, as female animals have historically been underrepresented in neuropathic pain research (33, 38, 45), while recent studies have emphasized the importance of incorporating sex as a biological variable in PBMT research (29, 32). By including both sexes and evaluating the influence of the estrous cycle, the present study contributes to a more comprehensive understanding of PBMT efficacy in neuropathic pain. Rather than supporting the assumption that identical irradiation parameters should necessarily be applied across sexes, our findings and previous experimental evidence from our group suggest that sex-specific optimization of PBMT may be warranted. Future studies should systematically investigate whether the sex-dependent optimization of PBMT parameters reflects differences in the biological mechanisms underlying photobiomodulation responsiveness. Direct comparisons of standardized and sex-optimized protocols may help determine whether differences in treatment efficacy arise from biological sex, differential sensitivity to specific irradiation parameters, or an interaction between these factors.

## Supplementary Information

Supplementary Material 1.

## Data Availability

Data are available from the authors upon reasonable request.
