# Supplementary material for: Sex-related analgesic responses to photobiomodulation therapy in rats with neuropathic pain
Source: Lasers Med Sci. 2026 Sep 25;41(1):242. doi: 10.1007/s10103-026-05046-5 (PMC13615017; doi:10.1007/s10103-026-05046-5)
Supplement: Supplementary file 1 [file 10103_2026_5046_MOESM1_ESM.docx]

| ***Laser Parameter*** | ***Female*** | ***Male*** |
| --- | --- | --- |
| **Start of treatment** | 14th day post-surgery | 14th day post-surgery |
| **Frequency** | 3 times per week | 3 times per week |
| **Total sessions** | 10 | 10 |
| **Wavelength** | 808nm- infrared | 904nm - infrared |
| **Emission mode** | Continuous | Continuous |
| **Power** | 100mW | 45mW |
| **Beam area** | 0,07cm² | 0,13cm² |
| **Energy density** | 57,14J/cm² | 6 J/cm² |
| **Energy per point** | 4J | 0,78J |
| **Number of points** | 7 points along the sciatic nerve | 9 points along the sciatic nerve |
| **Energy per session** | 28J | 7,02J |
| **Total treatment energy** | 280J | 70,2J |

**Table 1: Photobiomodulation parameters used in female and male animals.**
